# Difference in pulmonary permeability between indirect and direct acute respiratory distress syndrome assessed by the transpulmonary thermodilution technique

**DOI:** 10.1186/cc12037

**Published:** 2013-03-19

**Authors:** K Morisawa, Y Taira, M Yanai, Y Takamatu, S Kushimoto, S Fujitani

**Affiliations:** 1 St Marianna University, Kawasaki-Shi, Japan; 2 Tohoku University Graduate School of Medicine, Sendai-shi, Japan; 3 Tokyo Bay Urayasu/Ichikawa Medical Center, Urayasu-shi, Japan

## Introduction

ARDS is characterized by increased pulmonary capillary permeability secondary to diffuse alveolar inflammation and injury. Common risk factors can be classified into two groups: extrapulmonary causes (indirect etiologies: ARDSexp) or pulmonary causes (direct etiologies: ARDSp). There are few quantitative methods to distinguish between the differences in these two ARDS categories.

## Methods

A subanalysis of the trial by the PiCCO Pulmonary Edema Study (prospective, observational, multi-institutional study) in 23 ICUs of academic tertiary referral hospitals in Japan. All consecutive adult patients requiring mechanical ventilation with the diagnosis of ARDS were monitored by the transpulmonary thermodilution technique system (PiCCO; Pulsion Medical Systems, Munich, Germany) for 3 days: day 0, day 1 and day 2. The pulmonary vascular permeability index (PVPI), extravascular lung water index and intrathoracic blood volume index were measured concurrently. Three experts retrospectively determined the pathophysiological mechanism causing ARDS by patient history, clinical presentation, chest computed tomography and radiography. Patients were classified into two groups: patients with ARDS triggered by ARDSexp and ARDSp.

## Results

During the study period from March 2009 to August 2011, a total of 173 patients were assessed including 56 ARDSexp patients and 117 ARDSp patients, with the most common cause of ARDSexp secondary to sepsis (71%) and of ARDSp pneumonia (80%). The measurement of PVPI was significantly elevated in the ARDSp group on all days. There were no significant differences in mortality at 28 days, mechanical ventilation days, and hospital length-of-stay between the two groups, while the ARDSexp group seemed to be associated with prolonged mechanical ventilation days and hospital length-of-stay.

## Conclusion

This study suggests the existence of several differences in pathogenetic pathways and the degree of pulmonary permeability between patients with ARDSexp and ARDSp. We therefore believe that using the PVPI may provide us with timely quantitative information to make clinical decisions.

